# Correction: Epigenetic scores for the circulating proteome as tools for disease prediction

**DOI:** 10.7554/eLife.94481

**Published:** 2023-11-20

**Authors:** Danni A Gadd, Robert F Hillary, Daniel L McCartney, Shaza B Zaghlool, Anna J Stevenson, Yipeng Cheng, Chloe Fawns-Ritchie, Cliff Nangle, Archie Campbell, Robin Flaig, Sarah E Harris, Rosie M Walker, Liu Shi, Elliot M Tucker-Drob, Christian Gieger, Annette Peters, Melanie Waldenberger, Johannes Graumann, Allan F McRae, Ian J Deary, David J Porteous, Caroline Hayward, Peter M Visscher, Simon R Cox, Kathryn L Evans, Andrew M McIntosh, Karsten Suhre, Riccardo E Marioni

**Keywords:** Human

Gadd DA, Hillary RF, McCartney DL, Zaghlool SB, Stevenson AJ, Cheng Y, Fawns-Ritchie C, Nangle C, Campbell A, Flaig R, Harris SE, Walker RM, Shi L, Tucker-Drob EM, Gieger C, Peters A, Waldenberger M, Graumann J, McRae AF, Deary IJ, Porteous DJ, Hayward C, Visscher PM, Cox SR, Evans KL, McIntosh AM, Suhre K, Marioni RE. 2022. Epigenetic scores for the circulating proteome as tools for disease prediction. *eLife* **11**:e71802. doi: 10.7554/eLife.71802.

Published 13 January 2022

**[Details of correction]**

In the study, protein EpiScores are generated and Cox proportional hazards (PH) analyses are run to test associations between each score and 12 incident diseases in Generation Scotland. The disease cases were obtained through electronic health data linkage. The Generation Scotland research team have made us aware of an error regarding the disease linkage data that was used in the original work. This affected the lung cancer disease codes, which were accidentally enriched for both breast and lung cancer traits. We have corrected this, with incident lung cancer cases falling from 201 to 100 in the corrected data. We also noticed an issue regarding the ranking of diagnosis dates for disease codes, which affected time-to-event calculations for a small proportion of disease cases. This has also been corrected. We now report 130 protein EpiScore associations with incident disease rather than 137. The type 2 diabetes EpiScore associations still replicate those observed in the literature, with highly similar results to those observed previously. Three of the original lung cancer associations remain (G-CSF, HGF and EN-RAGE), whereas five are no longer statistically significant and seven new associations are observed. The revised Figures 4 and 6 show the updated Cox PH association results. All corresponding tables in Supplementary file 1 have also been updated to reflect the change in the Cox PH analyses. As it is only the Cox PH association analyses in Generation Scotland that has been updated, the protein EpiScore weights remain unchanged and are usable in future research contexts without need for amendment. The central message of our study (that EpiScores are useful tools for disease prediction) also remains unchanged.

The necessitated changes to the Cox PH sections of the work are outlined below. Underline indicates where updates to the corrected text passages have been made, versus the original text.

**[Abstract]**

[Corrected text:]

we uncovered 130 EpiScore – disease associations

[Original text:]

we uncovered 137 EpiScore – disease associations

**[eLife digest]**

[Corrected text:]

revealed 130 connections

[Original text:]

revealed 137 connections

[Removed text:]

The following text has been removed due to the video no longer reflecting the updated results:

A video summarising this study and detailing how the 109 EpiScores can be calculated in cohorts with DNAm data is available at: https://youtu.be/xKDWg0Wzvrg.

**[Figure 1:]**

[Corrected figure:]

**Figure 1:**
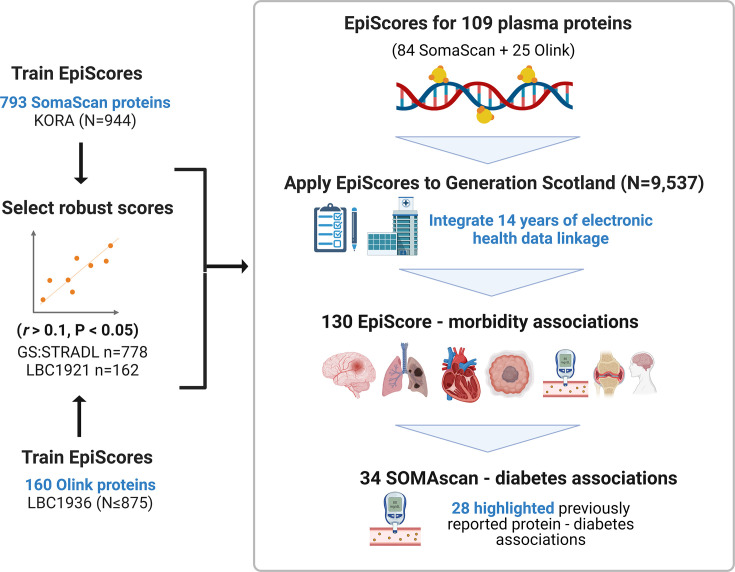


**Figure 1. EpiScores for plasma proteins as tools for disease prediction study design**. DNA methylation scores were trained on 953 circulating plasma protein levels in the KORA and LBC1936 cohorts. There were 109 EpiScores selected based on performance (*r*>0.1, *P*<0.05) in independent test sets. The selected EpiScores were projected into Generation Scotland, a cohort that has extensive data linkage to GP and hospital records. We tested whether levels of each EpiScore at baseline could predict the onset of 12 leading causes of morbidity, over a follow-up period of up to 14 years. 130 EpiScore – disease associations were identified, for 10 morbidities. We then assessed whether EpiScore associations reflected protein associations for diabetes, which is a trait that has been well-characterised using SOMAscan protein measurements. Of the 34 SOMAscan-derived EpiScore – diabetes associations, 28 highlighted previously reported protein - diabetes associations.

[Original figure:]

**Figure 2:**
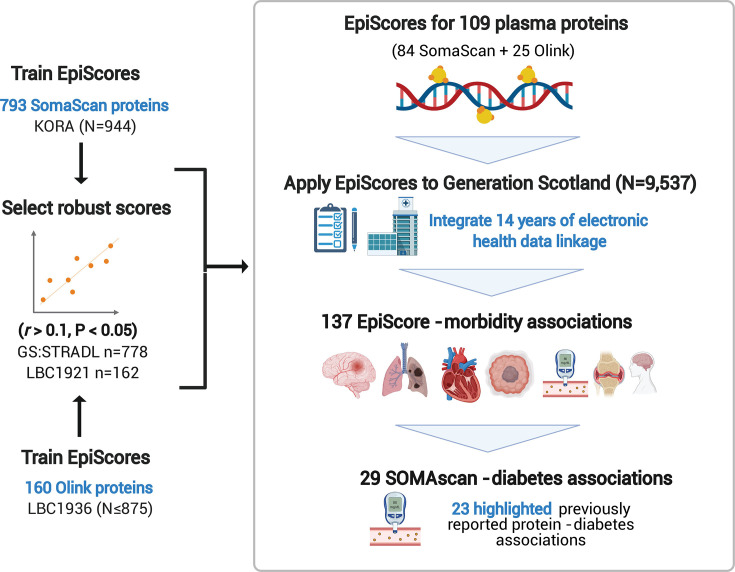


**Figure 1. EpiScores for plasma proteins as tools for disease prediction study design**. DNA methylation scores were trained on 953 circulating plasma protein levels in the KORA and LBC1936 cohorts. There were 109 EpiScores selected based on performance (*r*>0.1, *P*<0.05) in independent test sets. The selected EpiScores were projected into Generation Scotland, a cohort that has extensive data linkage to GP and hospital records. We tested whether levels of each EpiScore at baseline could predict the onset of 12 leading causes of morbidity, over a follow-up period of up to 14 years. 137 EpiScore – disease associations were identified, for 11 morbidities. We then assessed whether EpiScore associations reflected protein associations for diabetes, which is a trait that has been well-characterised using SOMAscan protein measurements. Of the 29 SOMAscan-derived EpiScore – diabetes associations, 23 highlighted previously reported protein - diabetes associations.

**[Table 1:]**

[Corrected table:]

**Table:** 

	Basic model			Fully-adjusted model		
**Morbidity**	**N cases**	**N Controls**	**Years to event (mean, sd**)	**N cases**	**N Controls**	**Years to event (mean, sd**)
Rheumatoid arthritis	63	9289	5.6 (3.5)	52	7742	6.1 (3.3)
Alzheimer’s disease	69	3764	7.7 (3)	52	3137	7.6 (3.1)
Bowel cancer	78	9398	6.4 (3.2)	66	7817	6.5 (3.2)
Depression	95	8317	4 (3.2)	75	6984	3.8 (3.2)
Lung cancer	100	9433	5.6 (3.2)	78	7850	5.6 (3.1)
Breast cancer	131	5356	6.1 (3.4)	111	4402	5.9 (3.4)
Inflammatory bowel disease	194	9114	5 (3.6)	155	7592	4.8 (3.6)
Stroke	313	9026	6.4 (3.4)	246	7547	6.3 (3.5)
COPD	322	8960	5.5 (3.4)	253	7476	5.5 (3.5)
Ischaemic heart disease	385	8649	5.6 (3.4)	302	7251	5.7 (3.4)
Diabetes	429	8757	5.6 (3.4)	322	7332	5.5 (3.4)
Pain	1329	5480	4.8 (3.5)	1081	4593	4.9 (3.5)

[Original table:]

**Table:** 

	Basic model			Fully-adjusted model		
**Morbidity**	**N cases**	**N Controls**	**Years to event (mean, sd**)	**N cases**	**N Controls**	**Years to event (mean, sd**)
Rheumatoid arthritis	65	9281	6.1 (3.5)	54	7736	6.4 (3.3)
Alzheimer’s dementia	69	3764	8.3 (2.7)	52	3137	8.2 (2.7)
Bowel cancer	77	9398	6.4 (3.2)	65	7817	6.5 (3.2)
Depression	101	8306	3.9 (3.3)	80	6976	3.7 (3.3)
Breast cancer	129	5355	6 (3.4)	110	4401	5.9 (3.4)
Lung cancer	201	9265	5.2 (3.1)	172	7705	5.1 (3.1)
Inflammatory bowel disease	203	9083	5 (3.5)	163	7567	4.9 (3.5)
Stroke	317	9023	6.5 (3.4)	248	7546	6.4 (3.5)
COPD	346	8939	6.2 (3.4)	273	7459	6.1 (3.4)
Ischaemic heart disease	395	8646	5.8 (3.3)	309	7248	5.9 (3.3)
Diabetes	428	8756	5.7 (3.4)	322	7331	5.7 (3.4)
Pain (back/neck)	1494	5341	5.2 (3.5)	1221	4475	5.3 (3.5)

**[Results: EpiScore-disease associations in Generation Scotland]**

[Corrected text:]

There were 286 EpiScore-disease associations with a False Discovery Rate (FDR)-adjusted *P*<0.05 in the basic model. After further adjustment for common risk factor covariates (smoking, social deprivation status, educational attainment, body mass index [BMI] and alcohol consumption), 130 of the 286 EpiScore-disease associations from the basic model had *P*<0.05 in the fully-adjusted model (**Supplementary files 1I-J**). Ten of the 130 fully-adjusted associations failed the Cox proportional hazards assumption for the EpiScore variable (*P*<0.05 for the association between the Schoenfeld residuals and time; **Supplementary file 1K**). When we restricted the time-to-event/censor period by each year of possible follow-up, there were minimal differences in the EpiScore-disease hazard ratios between follow-up periods that did not violate the assumption and those that did (**Supplementary file 1L**). The 130 associations were therefore retained as the primary results.

The 130 associations found in the fully-adjusted model comprised 70 unique EpiScores that were related to the incidence of 10 of the 12 morbidities studied. Diabetes and chronic obstructive pulmonary disease (COPD) had the greatest number of associations, with 38 and 37, respectively. **Figure 4** presents the EpiScore-disease relationships for COPD and the remaining nine morbidities: stroke, lung cancer, ischaemic heart disease (IHD), inflammatory bowel disease (IBD), rheumatoid arthritis (RA), depression, bowel cancer and pain (back/neck). There were 16 EpiScores that associated with the onset of three or more morbidities. **Figure 5** presents relationships for these 16 EpiScores in the fully-adjusted Cox model results. Of note is the EpiScore for Complement 5 (C5), which associated with four outcomes: stroke, diabetes, RA and COPD. Of the 34 SOMAscan-derived EpiScore associations with incident diabetes, 28 replicated previously reported protein associations (Elhadad et al., 2020; Gudmundsdottir et al., 2020; Ngo et al., 2021) with incident or prevalent diabetes in one or more cohorts (**Figure 6 and Supplementary file 1M**).

[Original text:]

Two associations in the basic model adjusting for age and sex failed to satisfy the global assumption (across all covariates) and were excluded. There were 294 remaining EpiScore-disease associations with a False Discovery Rate (FDR)-adjusted *P*<0.05 in the basic model. After further adjustment for common risk factor covariates (smoking, social deprivation status, educational attainment, body mass index [BMI] and alcohol consumption), 137 of the 294 EpiScore-disease associations from the basic model had *P*<0.05 in the fully-adjusted model (**Supplementary files 1I-J**). Eleven of the 137 fully-adjusted associations failed the Cox proportional hazards assumption for the EpiScore variable (*P*<0.05 for the association between the Schoenfeld residuals and time; **Supplementary file 1K**). When we restricted the time-to-event/censor period by each year of possible follow-up, there were minimal differences in the EpiScore - disease hazard ratios between follow-up periods that did not violate the assumption and those that did (**Supplementary file 1L**). The 137 associations were therefore retained as the primary results.

The 137 associations found in the fully-adjusted model comprised 78 unique EpiScores that were related to the incidence of 11 of the 12 morbidities studied. Diabetes and chronic obstructive pulmonary disease (COPD) had the greatest number of associations, with 33 and 41, respectively. **Figure 4** presents the EpiScore-disease relationships for COPD and the remaining nine morbidities: stroke, lung cancer, ischaemic heart disease (IHD), inflammatory bowel disease (IBD), rheumatoid arthritis (RA), depression, bowel cancer, pain (back/neck) and Alzheimer’s dementia. There were 13 EpiScores that associated with the onset of three or more morbidities. **Figure 5** presents relationships for these 13 EpiScores in the fully-adjusted Cox model results. Of note is the EpiScore for Complement 5 (C5), which associated with five outcomes: stroke, diabetes, IHD, RA and COPD. Of the 29 SOMAscan-derived EpiScore associations with incident diabetes, 23 replicated previously reported protein associations (Elhadad et al., 2020; Gudmundsdottir et al., 2020; Ngo et al., 2021) with incident or prevalent diabetes in one or more cohorts (**Figure 6 and Supplementary file 1M**).

**[Figure 3:]**

[Corrected figure:]

**Figure 3:**
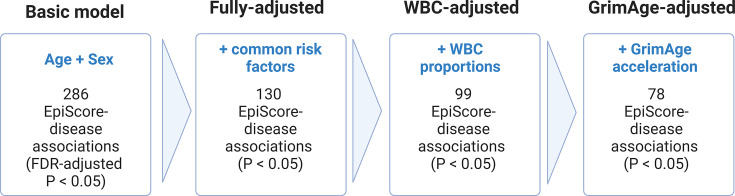


**Figure 3. Nested Cox proportional hazards assessment of protein EpiScore-disease prediction.** Mixed effects Cox proportional hazards analyses in Generation Scotland (n=9,537) tested the relationships between each of the 109 selected EpiScores and the incidence of 12 leading causes of morbidity (**Supplementary files 1I-J**). The basic model was adjusted for age and sex and yielded 286 associations between EpiScores and disease diagnoses, with false discovery rate (FDR)-adjusted *P*<0.05. In the fully-adjusted model, which included common risk factors as additional covariates (smoking, deprivation, educational attainment, body mass index (BMI) and alcohol consumption) 130 of the basic model associations remained significant with *P*<0.05. In a sensitivity analysis, the addition of estimated white blood cells (WBCs) to the fully-adjusted models led to the attenuation of 31 of the 130 associations. In a further sensitivity analysis, 78 associations remained after adjustment for both immune cell proportions and GrimAge acceleration.

[Original figure:]

**Figure 4:**
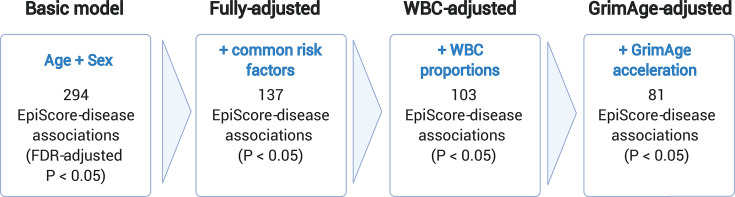


**Figure 3. Nested Cox proportional hazards assessment of protein EpiScore-disease prediction.** Mixed effects Cox proportional hazards analyses in Generation Scotland (n=9,537) tested the relationships between each of the 109 selected EpiScores and the incidence of 12 leading causes of morbidity (**Supplementary files 1I-J**). The basic model was adjusted for age and sex and yielded 294 associations between EpiScores and disease diagnoses, with false discovery rate (FDR)-adjusted *P*<0.05. In the fully-adjusted model, which included common risk factors as additional covariates (smoking, deprivation, educational attainment, body mass index (BMI) and alcohol consumption) 137 of the basic model associations remained significant with *P*<0.05. In a sensitivity analysis, the addition of estimated white blood cells (WBCs) to the fully-adjusted models led to the attenuation of 34 of the 137 associations. In a further sensitivity analysis, 81 associations remained after adjustment for both immune cell proportions and GrimAge acceleration.

**[Figure 4:]**

[Corrected figure:]

**Figure 5:**
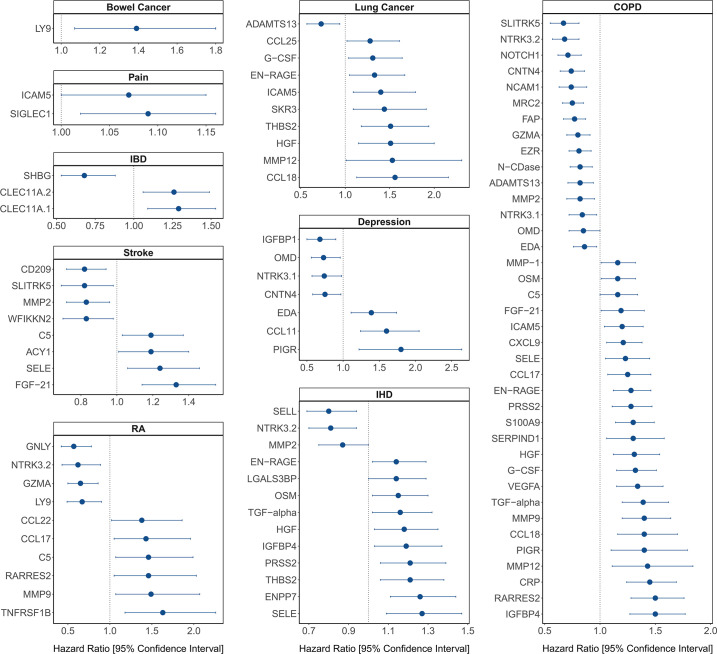


**Figure 4. Protein EpiScore associations with incident disease**. EpiScore-disease associations for 9 of the 11 morbidities with associations where *P*<0.05 in the fully-adjusted mixed effects Cox proportional hazards models in Generation Scotland (n=9,537). Hazard ratios are presented with confidence intervals for 92 of the 130 EpiScore-incident disease associations reported. Models were adjusted for age, sex and common risk factors (smoking, body mass index (BMI), alcohol consumption, deprivation and educational attainment). IBD: inflammatory bowel disease. IHD: ischaemic heart disease. COPD: chronic obstructive pulmonary disease. For EpiScore-diabetes associations, see **Figure 6**. Data shown corresponds to the results included in **Supplementary file 1J**.

[Original figure:]

**Figure 6:**
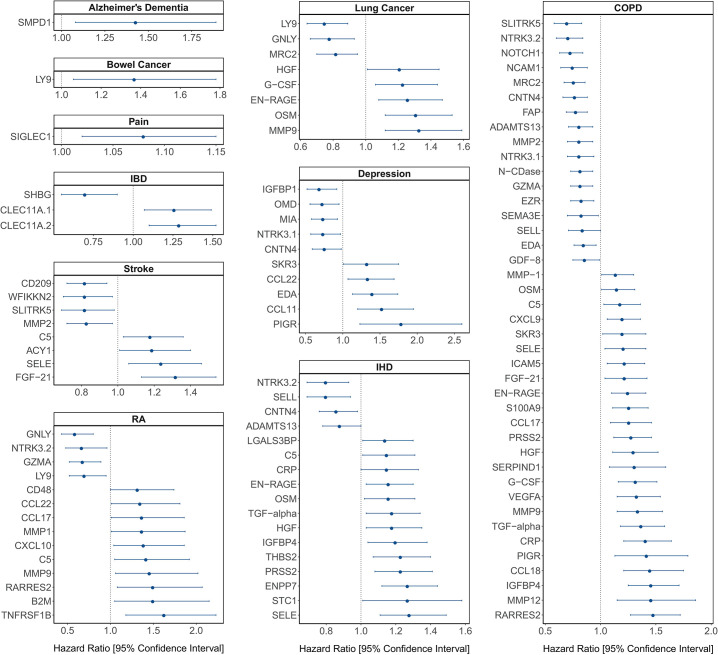


**Figure 4. Protein EpiScore associations with incident disease.** EpiScore-disease associations for ten of the eleven morbidities with associations where *P*<0.05 in the fully-adjusted mixed effects Cox proportional hazards models in Generation Scotland (n=9,537). Hazard ratios are presented with confidence intervals for 104 of the 137 EpiScore-incident disease associations reported. Models were adjusted for age, sex and common risk factors (smoking, body mass index (BMI), alcohol consumption, deprivation and educational attainment). IBD: inflammatory bowel disease. IHD: ischaemic heart disease. COPD: chronic obstructive pulmonary disease. For EpiScore-diabetes associations, see **Figure 6**. Data shown corresponds to the results included in **Supplementary file 1J**.

**[Figure 5:]**

[Corrected figure:]

**Figure 7:**
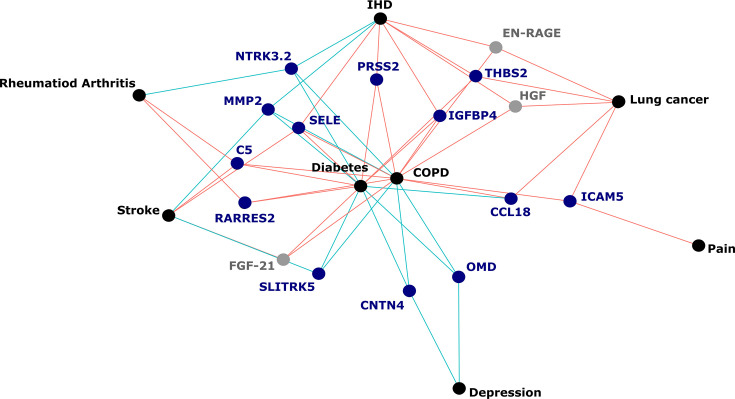


**Figure 5. Protein EpiScores that associated with the greatest number of morbidities.** EpiScores with a minimum of three relationships with incident morbidities in the fully-adjusted Cox models. The network includes 16 EpiScores as dark blue (SOMAscan) and grey (Olink) nodes, with disease outcomes in black. EpiScore-disease associations with hazard ratios <1 are shown as blue connections, whereas hazard ratios >1 are shown in red. COPD: chronic obstructive pulmonary disease. IHD: ischaemic heart disease. Data shown corresponds to the results included in **Supplementary file 1J**.

[Original figure:]

**Figure 8:**
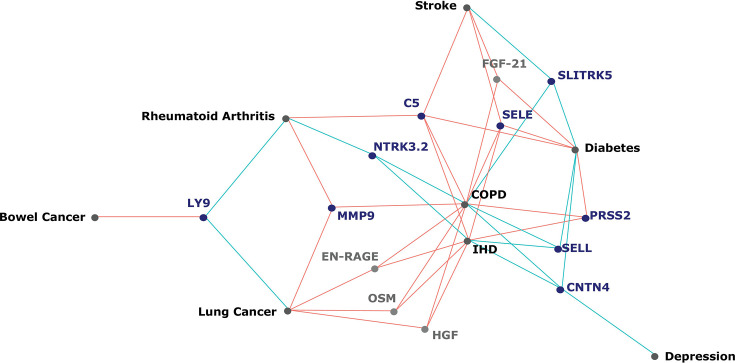


**Figure 5. Protein EpiScores that associated with the greatest number of morbidities.** EpiScores with a minimum of three relationships with incident morbidities in the fully-adjusted Cox models. The network includes 13 EpiScores as dark blue (SOMAscan) and grey (Olink) nodes, with disease outcomes in black. EpiScore-disease associations with hazard ratios <1 are shown as blue connections, whereas hazard ratios >1 are shown in red. COPD: chronic obstructive pulmonary disease. IHD: ischaemic heart disease. Data shown corresponds to the results included in **Supplementary file 1J**.

**[Figure 6:]**

[Corrected figure:]

**Figure 9:**
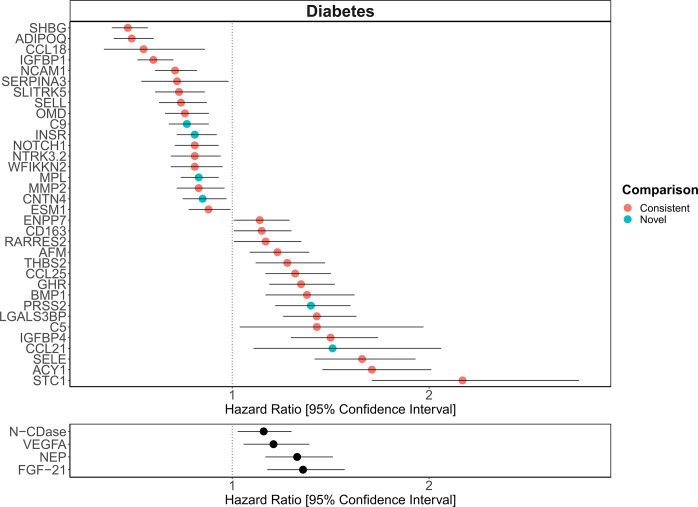


**Figure 6. Replication of known protein-diabetes associations with protein EpiScores**. EpiScore-incident diabetes associations in Generation Scotland (n=9,537). The 34 SOMAscan (top panel) and four Olink (bottom panel) associations shown with *P*<0.05 in fully-adjusted mixed effects Cox proportional hazards models. Of the 34 SOMAscan-derived EpiScores, 28 associations were consistent with protein-diabetes associations (pink) in one or more of the comparison studies that used SOMAscan protein levels. Six associations were novel (blue). Data shown corresponds to the results included in **Supplementary files 1J and 1M**.

[Original figure:]

**Figure 10:**
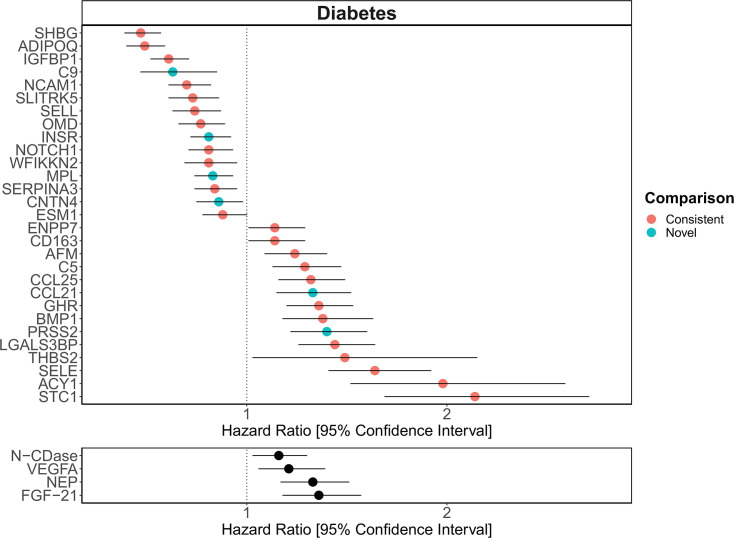


**Figure 6. Replication of known protein-diabetes associations with protein EpiScores**. EpiScore-incident diabetes associations in Generation Scotland (n=9,537). The 29 SOMAscan (top panel) and four Olink (bottom panel) associations shown with *P*<0.05 in fully-adjusted mixed effects Cox proportional hazards models. Of the 29 SOMAscan-derived EpiScores, 23 associations were consistent with protein-diabetes associations (pink) in one or more of the comparison studies that used SOMAscan protein levels. Six associations were novel (blue). Data shown corresponds to the results included in **Supplementary files 1J and 1M**.

**[Results: Immune cell and GrimAge sensitivity analyses]**

[Corrected text:]

Correlations of the 70 EpiScores that were associated with incident disease…

[Original text:]

Correlations of the 78 EpiScores that were associated with incident disease…

[Corrected text:]

…99 associations that remained statistically significant (FDR *P*<0.05 in the basic model and *P*<0.05 in the fully-adjusted model) after adjustment for immune cell proportions, of which 78 remained significant…

[Original text:]

…103 associations that remained statistically significant (FDR *P*<0.05 in the basic model and *P*<0.05 in the fully-adjusted model) after adjustment for immune cell proportions, of which 81 remained significant…

[Corrected text:]

Of the 60 possible relationships between WBC measures and the morbidities assessed, three were statistically significant (FDR-adjusted *P*<0.05)

[Original text:]

Of the 60 possible relationships between WBC measures and the morbidities assessed, four were statistically significant (FDR-adjusted *P*<0.05)

[Corrected text:]

A higher proportion of natural killer cells was linked to decreased risk of incident COPD, RA and diabetes. The GrimAge acceleration composite score was associated with COPD, lung cancer, IBD, diabetes and RA…

[Original text:]

A higher proportion of natural killer cells was linked to decreased risk of incident COPD, rheumatoid arthritis, diabetes and pain (back/neck). The GrimAge acceleration composite score was associated with COPD, IHD, diabetes and pain (back/neck)…

**[Discussion:]**

[Corrected text:]

we show that 70 EpiScores associate with the incidence of 10 leading causes of morbidity (130 EpiScore-disease associations in total)

[Original text:]

we show that 78 EpiScores associate with the incidence of 11 leading causes of morbidity (137 EpiScore-disease associations in total)

[Corrected text:]

top markers identified included aminoacylase-1 (ACY-1), sex hormone binding globulin (SHBG) and growth hormone receptor (GHR)

[Original text:]

top markers identified included aminoacylase-1 (ACY-1), sex hormone binding globulin (SHBG), growth hormone receptor (GHR) and insulin-like growth factor-binding protein 2 (IGFBP-2)

[Removed text:]

The following text has been removed as the contents are no longer relevant to the updated results:

For example, levels of the acid sphingomyelinase (ASM) EpiScore predicted onset of Alzheimer’s dementia, several years prior to diagnosis. ASM (encoded by SMPD1) has been discussed as a therapeutic candidate for Alzheimer’s disease (Cataldo et al., 2004; Lee et al., 2014; Park et al., 2020) and has been shown to disrupt autophagic protein degradation and associate with accumulation of amyloid-beta in murine models of Alzheimer’s pathology (Lee et al., 2014; Park et al., 2020).

[Corrected text:]

the EpiScore for Complement Component 5 (C5), which was associated with the onset of four morbidities

[Original text:]

the EpiScore for Complement Component 5 (C5), which was associated with the onset of five morbidities, the highest number for any EpiScore

**[Materials and methods:]**

[Corrected text:]

A false discovery rate multiple testing correction *P*<0.05 was applied to the 1308 EpiScore-disease associations (109 EpiScores by 12 incident disease traits)

[Original text:]

A false discovery rate multiple testing correction *P*<0.05 was applied to the 1306 EpiScore-disease associations (109 EpiScores by 12 incident disease traits, with 2 associations excluded for failing the global proportional hazards assumption).

**[Data availability:]**

[Removed text:]

The following text has been removed due to the video no longer reflecting the updated results:

A video summarising this study and detailing how the 109 EpiScores can be calculated in cohorts with DNAm data is available at: https://youtu.be/xKDWg0Wzvrg.

**[Supplementary file 1:]**

**Supplementary file 1** - 1J, 1K, 1L, 1M, 1N, 1O have been updated accordingly to reflect the new Cox PH analyses.

**[Figure 3—figure supplement 1. Phenotypic trait and estimated white blood cell proportion correlations with EpiScores:]**

[Corrected text in figure legend:]

Heatmap of Pearson’s correlations (*r*) between the 70 protein EpiScore measures for Olink proteins which were associated with incident disease…

[Original text in figure legend:]

Heatmap of Pearson’s correlations (*r*) between the 78 protein EpiScore measures for Olink proteins which were associated with incident disease…

[Corrected figure:]

**Figure 11:**
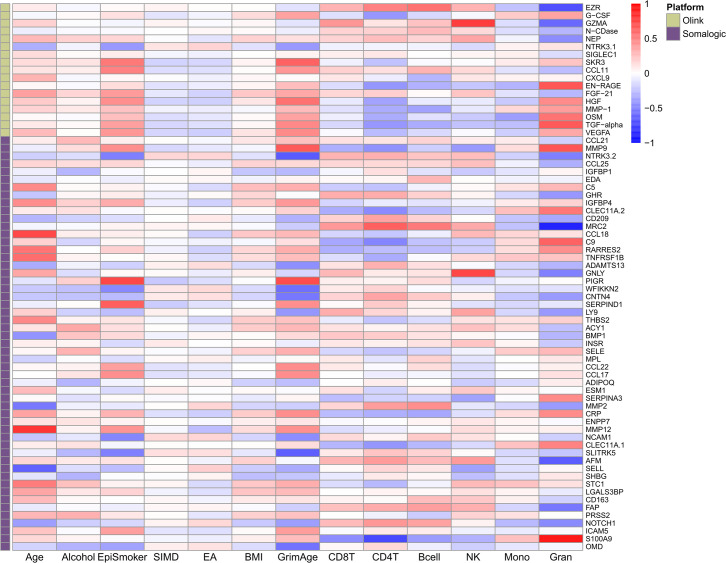


[Original figure:]

**Figure 12:**
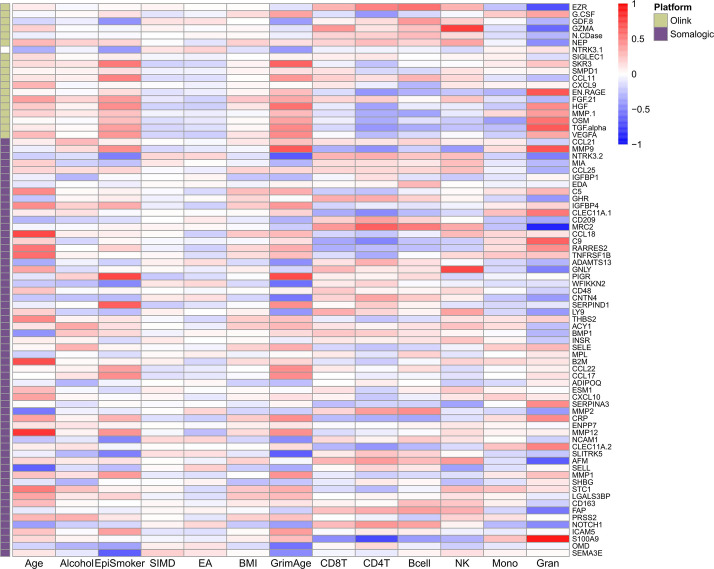


The article, supplementary file 1 and figures have been corrected accordingly.

