# Supplementary material for: Epigenetic scores for the circulating proteome as tools for disease prediction
Source: eLife. 2022 Jan 13;11:e71802. doi: 10.7554/eLife.71802 (PMC8880990; doi:10.7554/eLife.71802)
Supplement: Transparent reporting form [file elife-71802-transrepform1.pdf]

1st Floor
24 Hills Road
Cambridge CB2 1JP, UK

P 01223 855340
W elifesciences.org
T @elife

eLife's transparent reporting form

We encourage authors to provide detailed information *within their submission* to facilitate the interpretation and replication of experiments. Authors can upload supporting documentation to indicate the use of appropriate reporting guidelines for health-related research (see EQUATOR Network), life science research (see the BioSharing Information Resource), or the ARRIVE guidelines for reporting work involving animal research. Where applicable, authors should refer to any relevant reporting standards documents in this form.

If you have any questions, please consult our Journal Policies and/or contact us: editorial@elifesciences.org.

Sample-size estimation

- You should state whether an appropriate sample size was computed when the study was being designed
- You should state the statistical method of sample size computation and any required assumptions
- If no explicit power analysis was used, you should describe how you decided what sample (replicate) size (number) to use

Please outline where this information can be found within the submission (e.g., sections or figure legends), or explain why this information doesn't apply to your submission:

Sample size was determined by selecting the maximum sample available for each cohort component of the study. All individuals with relevant methylation, genetic or proteomic data were included. This information is specified in the sample population and cohort-specific sections of our methods. The datasets we utilize represent two of the largest populations with DNA methylation and protein data for training of SOMAscan EpiScores that exist globally (i.e. the KORA and STRADL cohorts, which had a total of 793 overlapping SOMAmers).

Replicates

- You should report how often each experiment was performed
- You should include a definition of biological versus technical replication
- The data obtained should be provided and sufficient information should be provided to indicate the number of independent biological and/or technical replicates
- If you encountered any outliers, you should describe how these were handled
- Criteria for exclusion/inclusion of data should be clearly stated
- High-throughput sequence data should be uploaded before submission, with a private link for reviewers provided (these are available from both GEO and ArrayExpress)

Please outline where this information can be found within the submission (e.g., sections or figure legends), or explain why this information doesn't apply to your submission:

eLife Sciences Publications, Ltd is a limited liability non-profit non-stock corporation incorporated in the State of Delaware, USA, with company number 5030732, and is registered in the UK with company number FC030576 and branch number BR015634 at the address 1st Floor, 24 Hills Road, Cambridge CB2 1JP | August 2014

Our study involves training and testing of protein EpiScores across several independent cohorts. Each protein level measurement was trained in one cohort and projected into a separate cohort for testing. In some instances, we were able to test the Olink protein measurements in two test cohorts (STRADL and LBC1921 for neurology proteins). We then projected 109 EpiScores that were selected based on their performance in the testing phase of the study into another cohort, Generation Scotland. We tested for relationships between the EpiScores for proteins and incident diseases. We did not include replication cohorts during this stage of the study, as we are unaware of a cohort that has the depth of profiling for electronic health data linkage to the morbidities we study, in addition to methylation data for projection of EpiScores. Outlying values in protein and EpiScore levels were addressed through rank-inverse based normalization of the original protein and projected EpiScore levels. This was conducted prior to running both the training/testing of EpiScores and the incident disease modelling with EpiScores. All individuals with the required data measurements available (i.e. genetic, epigenetic or proteomic) were included in the study and this is detailed in the sample population sections of the methods. Data from the cohorts we study involve human participants and therefore can be accessed by contacting the cohort administrators. We have provided full details for each of the cohorts used in our manuscript file.

Statistical reporting

- Statistical analysis methods should be described and justified
- Raw data should be presented in figures whenever informative to do so (typically when N per group is less than 10)
- For each experiment, you should identify the statistical tests used, exact values of N, definitions of center, methods of multiple test correction, and dispersion and precision measures (e.g., mean, median, SD, SEM, confidence intervals; and, for the major substantive results, a measure of effect size (e.g., Pearson's r, Cohen's d)
- Report exact p-values wherever possible alongside the summary statistics and 95% confidence intervals. These should be reported for all key questions and not only when the p-value is less than 0.05.

Please outline where this information can be found within the submission (e.g., sections or figure legends), or explain why this information doesn't apply to your submission:

All statistical analyses are described in our methods section and full results for each test performed are available in our Supplemental data. All N's for each cohort available are provided in Supplementary file 1A. N's for specific statistical tests are provided for each set of results in Figure legends and in Supplemental data documenting the results. Briefly, Pearson's, two-sided correlation coefficients (*r*) were extracted to compare projected EpiScores with test set protein levels and are presented in Figure 2 and Supplementary files 1B-C. We then chose Cox proportional hazard models to test for associations between protein EpiScores and incident diseases in Generation Scotland, as this approach can be used to adjust for a range of covariates and relatedness between individuals when assessing time-to-event. Hazard ratios and standard errors are extracted from these associations and are documented in Supplementary files 1I-M. These results form the basis of Figures 4-6. Exact values and coefficients are reported in all cases. For all sensitivity analyses and analyses with COVID-19 outcomes, full statistical tables are reported in Supplementary files 1N-P.

(For large datasets, or papers with a very large number of statistical tests, you may upload a single table file with tests, Ns, etc., with reference to sections in the manuscript.)

Group allocation

- Indicate how samples were allocated into experimental groups (in the case of clinical studies, please specify allocation to treatment method); if randomization was used, please also state if restricted randomization was applied
- Indicate if masking was used during group allocation, data collection and/or data analysis

Please outline where this information can be found within the submission (e.g., sections or figure legends), or explain why this information doesn't apply to your submission:

There were no experimental, randomized groups allocated in this study and no treatment methods were used.

All available individuals for training and testing protein EpiScores were included in our initial training and validation of EpiScores. Retrospective electronic health data linkage was then used to identify those individuals who were cases for each of the 12 morbidities we assessed in relation to the projected levels of protein EpiScores at baseline. The diagnoses codes used to classify cases and controls are listed in Supplementary file 1 for each morbidity. A summary of case/control inclusion details can also be found in the methods section that details Cox proportional hazards modelling, with more details provided in the methods section. All possible cases of COVID-19 hospitalisation and long-COVID were also included in the COVID-19 analyses.

Additional data files ("source data")

- We encourage you to upload relevant additional data files, such as numerical data that are represented as a graph in a figure, or as a summary table
- Where provided, these should be in the most useful format, and they can be uploaded as "Source data" files linked to a main figure or table
- Include model definition files including the full list of parameters used
- Include code used for data analysis (e.g., R, MatLab)
- Avoid stating that data files are "available upon request"

Please indicate the figures or tables for which source data files have been provided:

To facilitate replication of our results and to allow for comparisons of our results to studies in future, we make full tables of our results available. Datasets generated in this study are made available in Supplementary file 1; this file includes the protein EpiScore weights for the 109 EpiScores we provide for future studies to use. All results datasets used to create figures are included in Supplementary file 1 and specific locations for these are noted in figure legends and provided here as follows: Figure 2: **Supplementary files 1B-C**, Figures 3-4**:** **Supplementary files 1J**, Figure 5: **Supplementary files 1J and 1M**.

All code used in the analyses is available with open access at the following Gitlab repository: https://github.com/DanniGadd/EpiScores-for-protein-levels.

The source datasets from the cohorts that were analysed during the current study are not publicly available due to them containing information that could compromise participant consent and confidentiality. Data can be obtained from the data owners. Instructions for Lothian Birth Cohort data access can be found here: https://www.lothianbirthcohort.ed.ac.uk/content/collaboration. Dr Simon Cox must be contacted to obtain a Lothian Birth Cohort 'Data Request Form' by email: simon.cox@ed.ac.uk. Instructions for accessing Generation Scotland data can be found here: https://www.ed.ac.uk/generation-scotland/for-researchers/access; the 'GS Access Request Form' can be downloaded from this site. Completed request forms must be sent to access@generationscotland.org to be approved by the Generation Scotland access committee. Data from the KORA study can be requested from KORA-gen: http://epi.helmholtz-muenchen.de/kora-gen. Requests are submitted online and are subject to approval by the KORA board.
